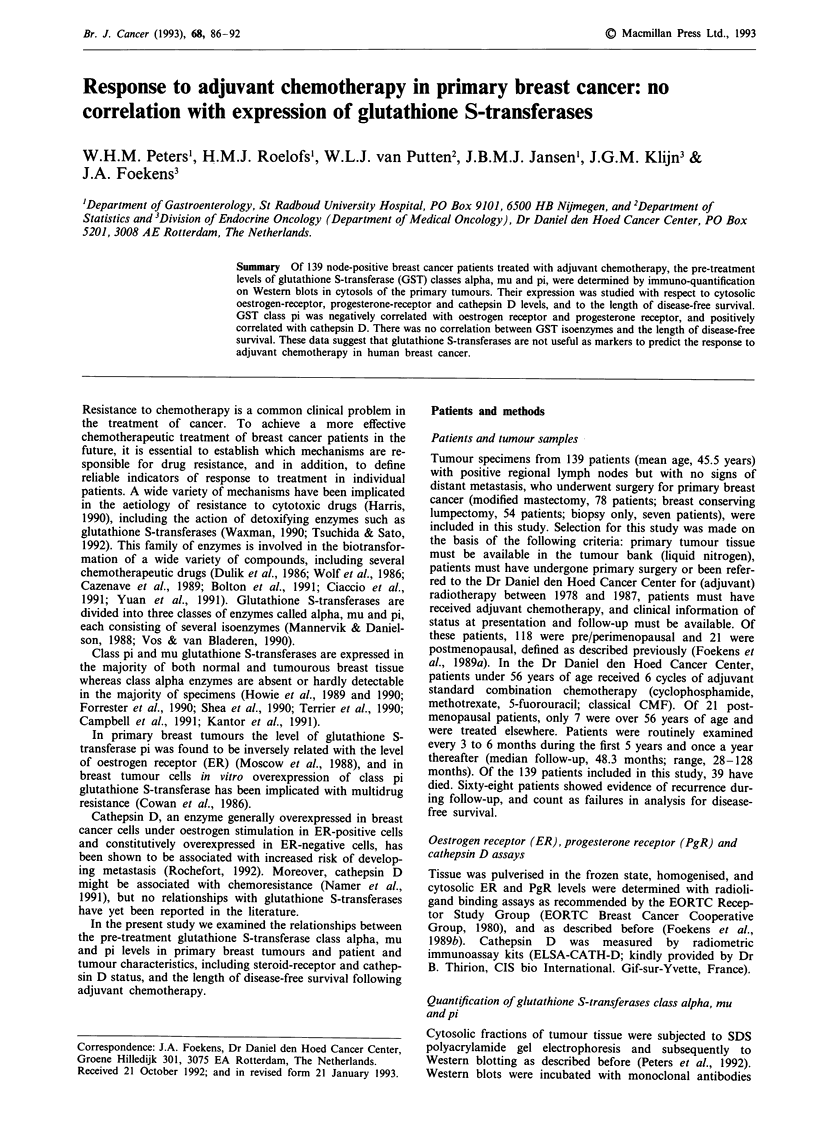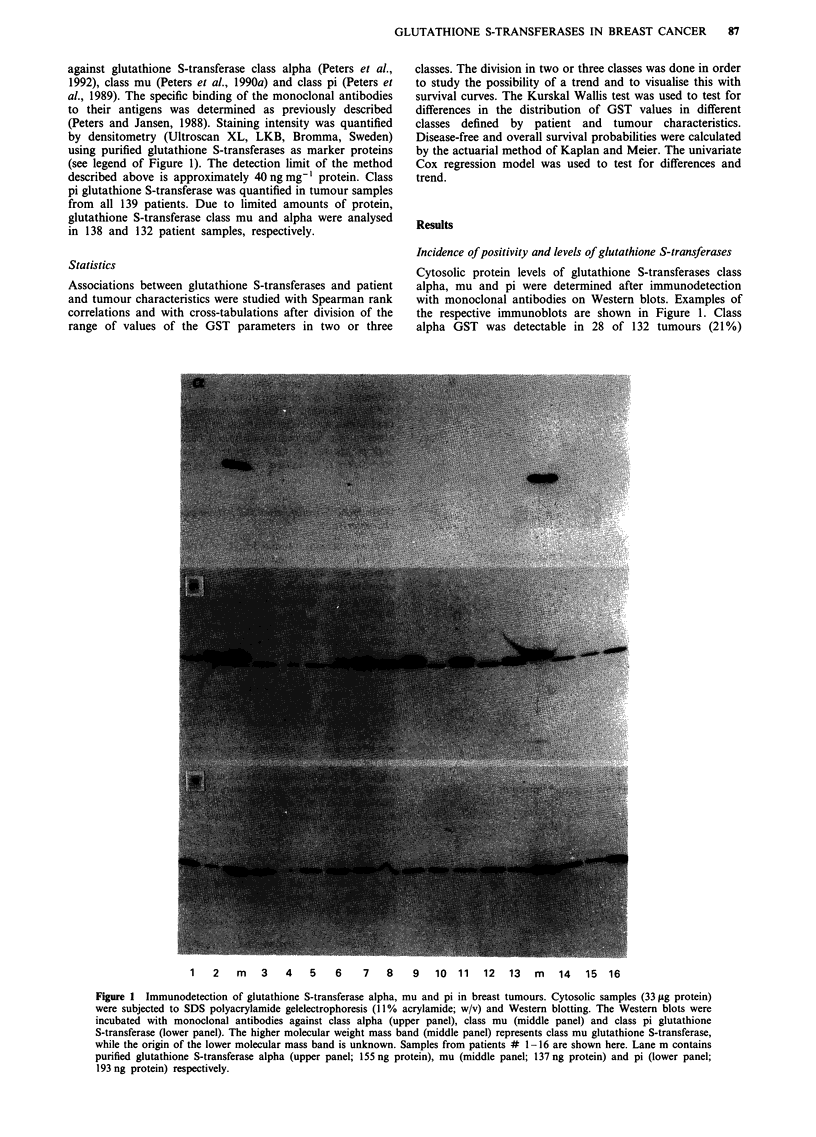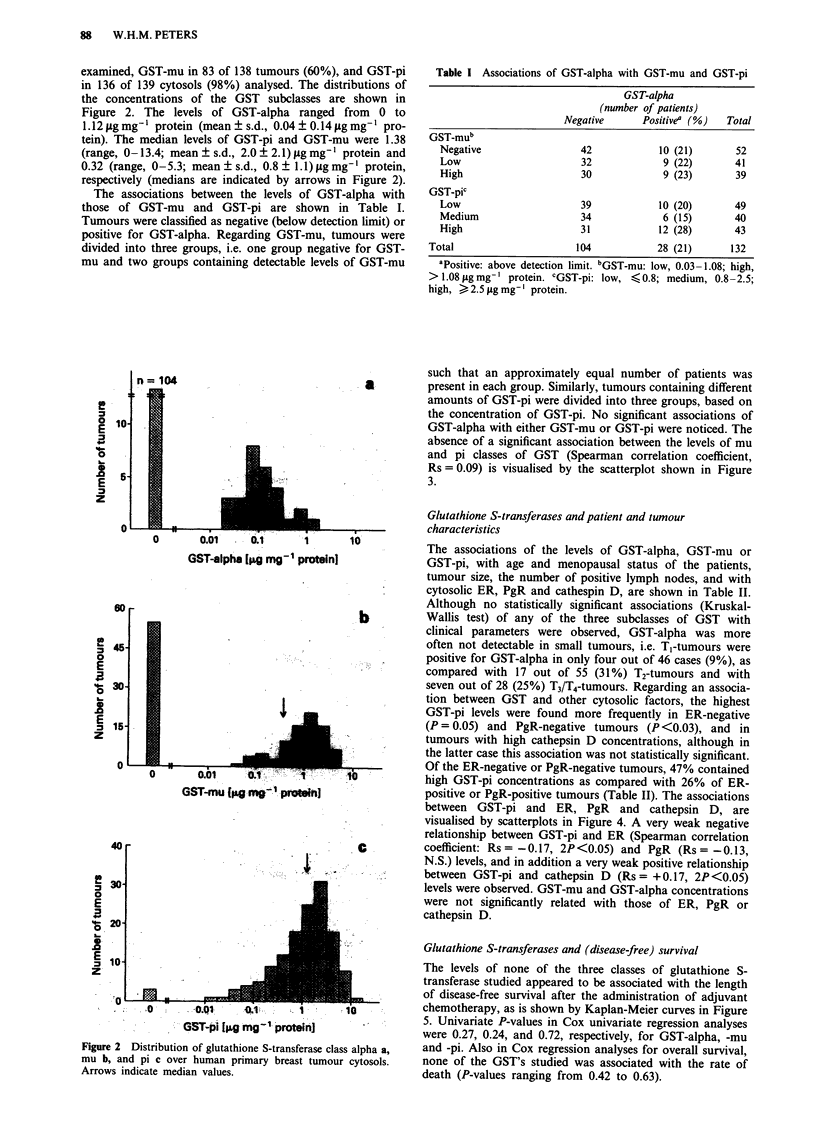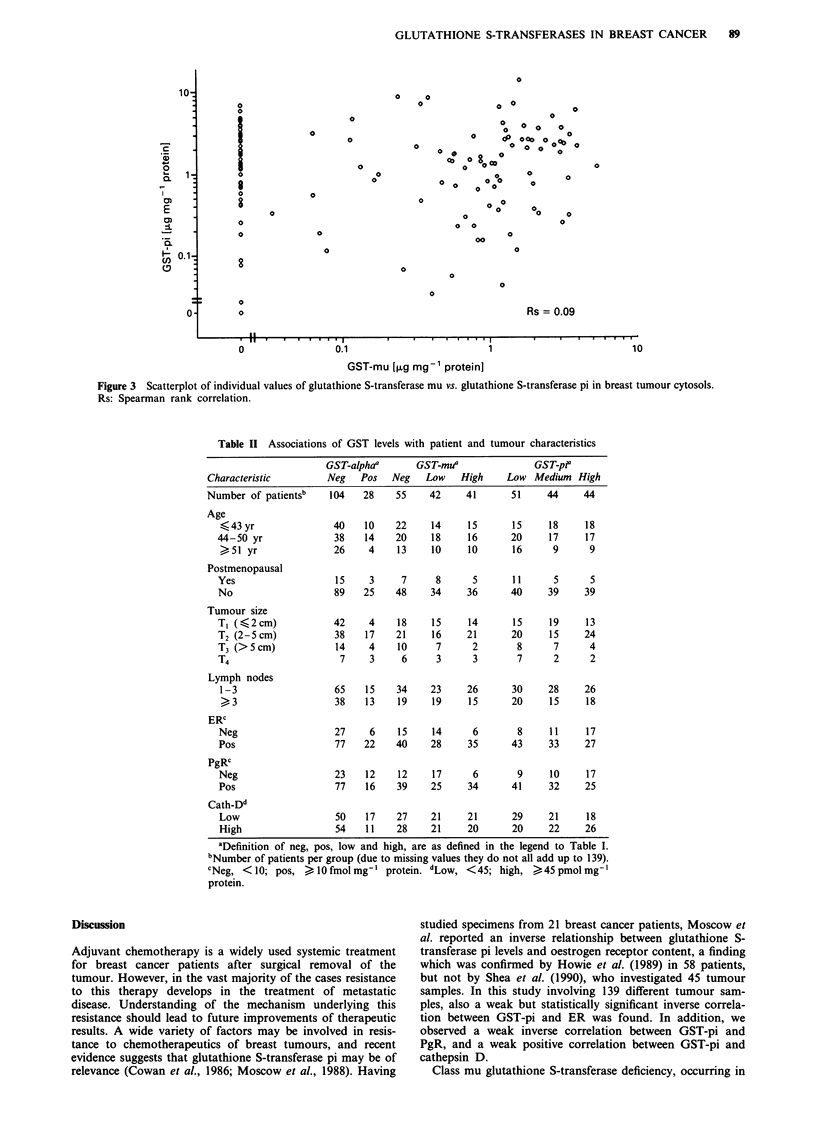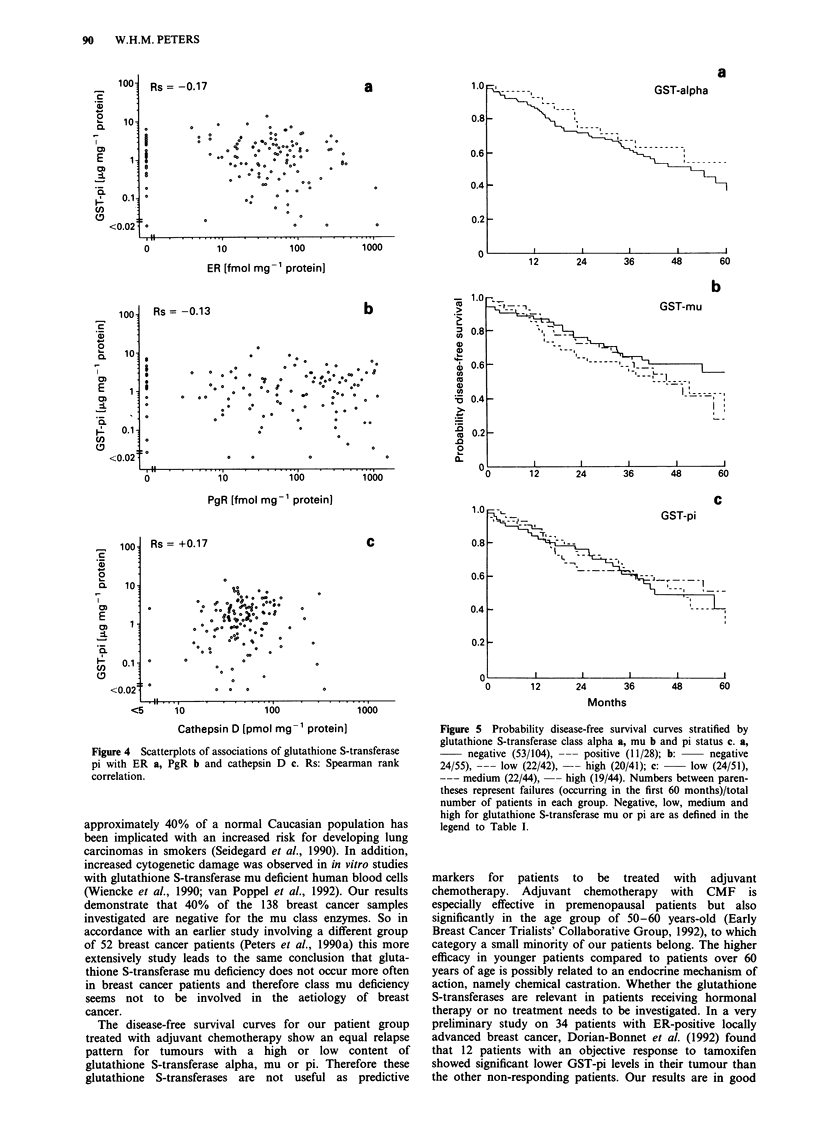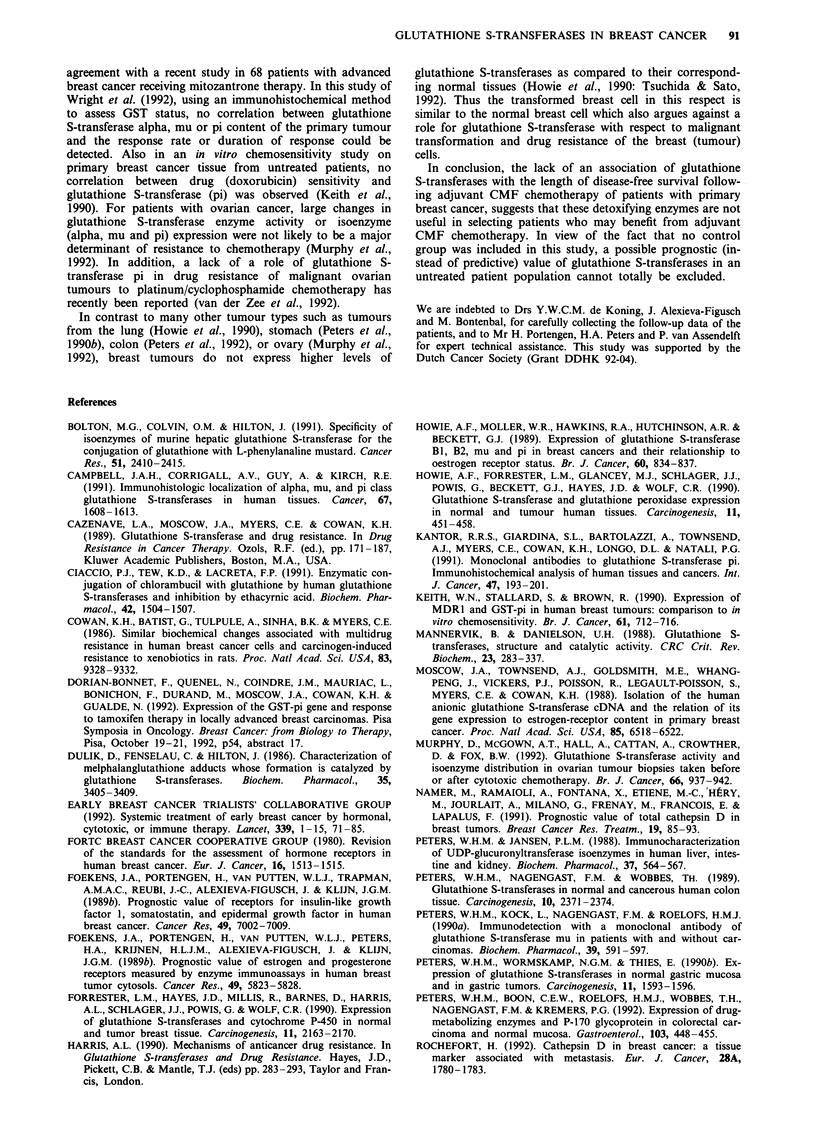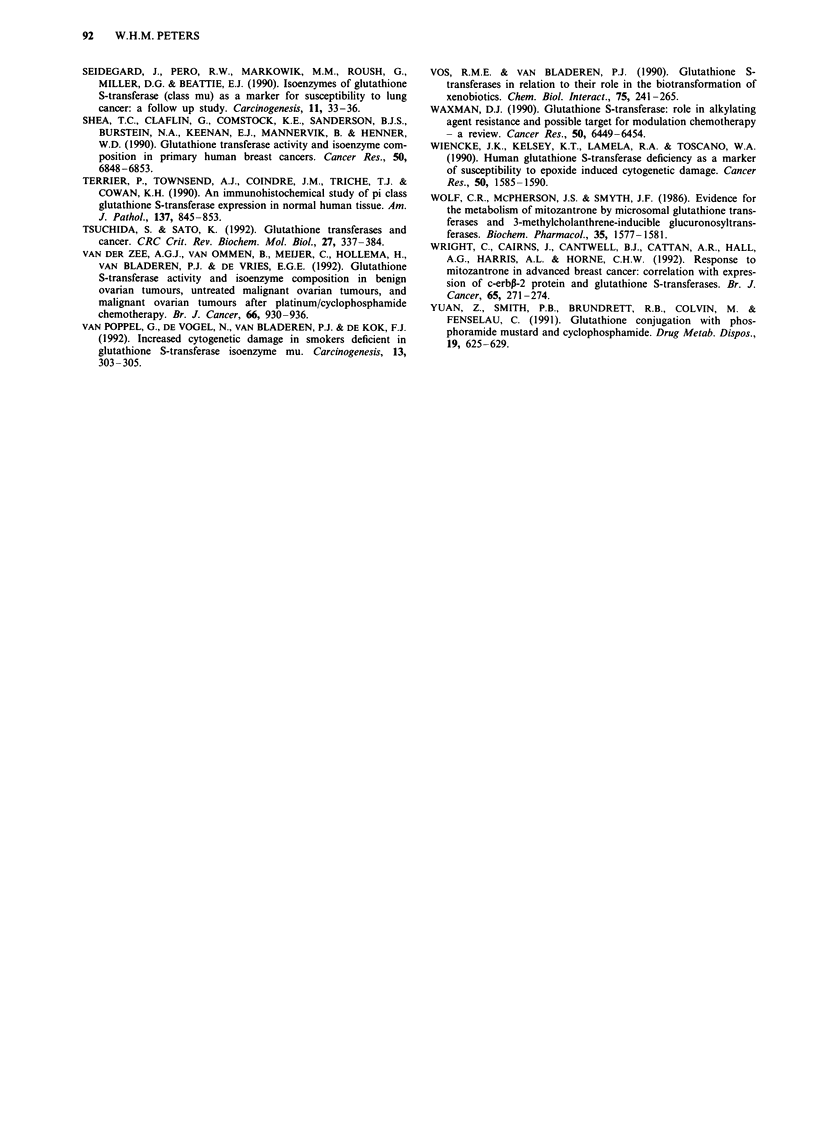# Response to adjuvant chemotherapy in primary breast cancer: no correlation with expression of glutathione S-transferases.

**DOI:** 10.1038/bjc.1993.291

**Published:** 1993-07

**Authors:** W. H. Peters, H. M. Roelofs, W. L. van Putten, J. B. Jansen, J. G. Klijn, J. A. Foekens

**Affiliations:** Department of Gastroenterology, St Radboud University Hospital, Nijmegen, The Netherlands.

## Abstract

**Images:**


Br. J. Cancer (1993), 68, 86-92 © Macmillan Press Ltd., 1993

### Response to adjuvant chemotherapy in primary breast cancer: no correlation with expression of glutathione S-transferases

W.H.M. Peters[1], H.M.J. Roelofs[1], W.L.J. van Putten[2], J.B.M.J. Jansen[1], J.G.M. Klijn[3] & J.A. Foekens[3]

[1]*Department of Gastroenterology, St Radboud University Hospital, PO Box 9101, 6500 HB Nijmegen, and* [2]*Department of Statistics and* [3]*Division of Endocrine Oncology (Department of Medical Oncology), Dr Daniel den Hoed Cancer Center, PO Box 5201, 3008 AE Rotterdam, The Netherlands.*

**Summary** Of 139 node-positive breast cancer patients treated with adjuvant chemotherapy, the pre-treatment levels of glutathione S-transferase (GST) classes alpha, mu and pi, were determined by immuno-quantification on Western blots in cytosols of the primary tumours. Their expression was studied with respect to cytosolic oestrogen-receptor, progesterone-receptor and cathepsin D levels, and to the length of disease-free survival. GST class pi was negatively correlated with oestrogen receptor and progesterone receptor, and positively correlated with cathepsin D. There was no correlation between GST isoenzymes and the length of disease-free survival. These data suggest that glutathione S-transferases are not useful as markers to predict the response to adjuvant chemotherapy in human breast cancer.

Resistance to chemotherapy is a common clinical problem in the treatment of cancer. To achieve a more effective chemotherapeutic treatment of breast cancer patients in the future, it is essential to establish which mechanisms are responsible for drug resistance, and in addition, to define reliable indicators of response to treatment in individual patients. A wide variety of mechanisms have been implicated in the aetiology of resistance to cytotoxic drugs (Harris, 1990), including the action of detoxifying enzymes such as glutathione S-transferases (Waxman, 1990; Tsuchida & Sato, 1992). This family of enzymes is involved in the biotransformation of a wide variety of compounds, including several chemotherapeutic drugs (Dulik *et al.*, 1986; Wolf *et al.*, 1986; Cazenave *et al.*, 1989; Bolton *et al.*, 1991; Ciaccio *et al.*, 1991; Yuan *et al.*, 1991). Glutathione S-transferases are divided into three classes of enzymes called alpha, mu and pi, each consisting of several isoenzymes (Mannervik & Danielson, 1988; Vos & van Bladeren, 1990).

Class pi and mu glutathione S-transferases are expressed in the majority of both normal and tumourous breast tissue whereas class alpha enzymes are absent or hardly detectable in the majority of specimens (Howie *et al.*, 1989 and 1990; Forrester *et al.*, 1990; Shea *et al.*, 1990; Terrier *et al.*, 1990; Campbell *et al.*, 1991; Kantor *et al.*, 1991).

In primary breast tumours the level of glutathione S-transferase pi was found to be inversely related with the level of oestrogen receptor (ER) (Moscow *et al.*, 1988), and in breast tumour cells *in vitro* overexpression of class pi glutathione S-transferase has been implicated with multidrug resistance (Cowan *et al.*, 1986).

Cathepsin D, an enzyme generally overexpressed in breast cancer cells under oestrogen stimulation in ER-positive cells and constitutively overexpressed in ER-negative cells, has been shown to be associated with increased risk of developing metastasis (Rochefort, 1992). Moreover, cathepsin D might be associated with chemoresistance (Namer *et al.*, 1991), but no relationships with glutathione S-transferases have yet been reported in the literature.

In the present study we examined the relationships between the pre-treatment glutathione S-transferase class alpha, mu and pi levels in primary breast tumours and patient and tumour characteristics, including steroid-receptor and cathepsin D status, and the length of disease-free survival following adjuvant chemotherapy.

Correspondence: J.A. Foekens, Dr Daniel den Hoed Cancer Center, Groene Hilledijk 301, 3075 EA Rotterdam, The Netherlands.
Received 21 October 1992; and in revised form 21 January 1993.

### Patients and methods

### *Patients and tumour samples*

Tumour specimens from 139 patients (mean age, 45.5 years) with positive regional lymph nodes but with no signs of distant metastasis, who underwent surgery for primary breast cancer (modified mastectomy, 78 patients; breast conserving lumpectomy, 54 patients; biopsy only, seven patients), were included in this study. Selection for this study was made on the basis of the following criteria: primary tumour tissue must be available in the tumour bank (liquid nitrogen), patients must have undergone primary surgery or been referred to the Dr Daniel den Hoed Cancer Center for (adjuvant) radiotherapy between 1978 and 1987, patients must have received adjuvant chemotherapy, and clinical information of status at presentation and follow-up must be available. Of these patients, 118 were pre/perimenopausal and 21 were postmenopausal, defined as described previously (Foekens *et al.*, 1989*a*). In the Dr Daniel den Hoed Cancer Center, patients under 56 years of age received 6 cycles of adjuvant standard combination chemotherapy (cyclophosphamide, methotrexate, 5-fuorouracil; classical CMF). Of 21 postmenopausal patients, only 7 were over 56 years of age and were treated elsewhere. Patients were routinely examined every 3 to 6 months during the first 5 years and once a year thereafter (median follow-up, 48.3 months; range, 28-128 months). Of the 139 patients included in this study, 39 have died. Sixty-eight patients showed evidence of recurrence during follow-up, and count as failures in analysis for disease-free survival.

### *Oestrogen receptor (ER), progesterone receptor (PgR) and cathepsin D assays*

Tissue was pulverised in the frozen state, homogenised, and cytosolic ER and PgR levels were determined with radioligand binding assays as recommended by the EORTC Receptor Study Group (EORTC Breast Cancer Cooperative Group, 1980), and as described before (Foekens *et al.*, 1989*b*). Cathepsin D was measured by radiometric immunoassay kits (ELSA-CATH-D; kindly provided by Dr B. Thirion, CIS bio International. Gif-sur-Yvette, France).

### *Quantification of glutathione S-transferases class alpha, mu and pi*

Cytosolic fractions of tumour tissue were subjected to SDS polyacrylamide gel electrophoresis and subsequently to Western blotting as described before (Peters *et al.*, 1992). Western blots were incubated with monoclonal antibodies against glutathione S-transferase class alpha (Peters *et al.*, 1992), class mu (Peters *et al.*, 1990*a*) and class pi (Peters *et al.*, 1989). The specific binding of the monoclonal antibodies to their antigens was determined as previously described (Peters and Jansen, 1988). Staining intensity was quantified by densitometry (Ultroscan XL, LKB, Bromma, Sweden) using purified glutathione S-transferases as marker proteins (see legend of Figure 1). The detection limit of the method described above is approximately 40 ng $mg^{-1}$ protein. Class pi glutathione S-transferase was quantified in tumour samples from all 139 patients. Due to limited amounts of protein, glutathione S-transferase class mu and alpha were analysed in 138 and 132 patient samples, respectively.

### *Statistics*

Associations between glutathione S-transferases and patient and tumour characteristics were studied with Spearman rank correlations and with cross-tabulations after division of the range of values of the GST parameters in two or three classes. The division in two or three classes was done in order to study the possibility of a trend and to visualise this with survival curves. The Kurskal Wallis test was used to test for differences in the distribution of GST values in different classes defined by patient and tumour characteristics. Disease-free and overall survival probabilities were calculated by the actuarial method of Kaplan and Meier. The univariate Cox regression model was used to test for differences and trend.

### Results

### *Incidence of positivity and levels of glutathione S-transferases*

Cytosolic protein levels of glutathione S-transferases class alpha, mu and pi were determined after immunodetection with monoclonal antibodies on Western blots. Examples of the respective immunoblots are shown in Figure 1. Class alpha GST was detectable in 28 of 132 tumours (21%)

**Figure 1** Immunodetection of glutathione S-transferase alpha, mu and pi in breast tumours. Cytosolic samples (33 μg protein) were subjected to SDS polyacrylamide gelelectrophoresis (11% acrylamide; w/v) and Western blotting. The Western blots were incubated with monoclonal antibodies against class alpha (upper panel), class mu (middle panel) and class pi glutathione S-transferase (lower panel). The higher molecular weight mass band (middle panel) represents class mu glutathione S-transferase, while the origin of the lower molecular mass band is unknown. Samples from patients # 1–16 are shown here. Lane m contains purified glutathione S-transferase alpha (upper panel; 155 ng protein), mu (middle panel; 137 ng protein) and pi (lower panel; 193 ng protein) respectively.

examined, GST-mu in 83 of 138 tumours (60%), and GST-pi in 136 of 139 cytosols (98%) analysed. The distributions of the concentrations of the GST subclasses are shown in Figure 2. The levels of GST-alpha ranged from 0 to 1.12 μg $mg^{-1}$ protein (mean ± s.d., 0.04 ± 0.14 μg $mg^{-1}$ protein). The median levels of GST-pi and GST-mu were 1.38 (range, 0–13.4; mean ± s.d., 2.0 ± 2.1) μg $mg^{-1}$ protein and 0.32 (range, 0–5.3; mean ± s.d., 0.8 ± 1.1) μg $mg^{-1}$ protein, respectively (medians are indicated by arrows in Figure 2).

The associations between the levels of GST-alpha with those of GST-mu and GST-pi are shown in Table I. Tumours were classified as negative (below detection limit) or positive for GST-alpha. Regarding GST-mu, tumours were divided into three groups, i.e. one group negative for GST-mu and two groups containing detectable levels of GST-mu such that an approximately equal number of patients was present in each group. Similarly, tumours containing different amounts of GST-pi were divided into three groups, based on the concentration of GST-pi. No significant associations of GST-alpha with either GST-mu or GST-pi were noticed. The absence of a significant association between the levels of mu and pi classes of GST (Spearman correlation coefficient, Rs = 0.09) is visualised by the scatterplot shown in Figure 3.

**Table I** Associations of GST-alpha with GST-mu and GST-pi

| | *GST-alpha (number of patients)* | | |
|---|---|---|---|
| | *Negative* | *Positive*[a] *(%)* | *Total* |
| GST-mu[b] | | | |
| Negative | 42 | 10 (21) | 52 |
| Low | 32 | 9 (22) | 41 |
| High | 30 | 9 (23) | 39 |
| GST-pi[c] | | | |
| Low | 39 | 10 (20) | 49 |
| Medium | 34 | 6 (15) | 40 |
| High | 31 | 12 (28) | 43 |
| Total | 104 | 28 (21) | 132 |

[a]Positive: above detection limit. [b]GST-mu: low, 0.03–1.08; high, > 1.08 μg $mg^{-1}$ protein. [c]GST-pi: low, ⩽0.8; medium, 0.8–2.5; high, ⩾2.5 μg $mg^{-1}$ protein.

**Figure 2** Distribution of glutathione S-transferase class alpha **a**, mu **b**, and pi **c** over human primary breast tumour cytosols. Arrows indicate median values.

### *Glutathione S-transferases and patient and tumour characteristics*

The associations of the levels of GST-alpha, GST-mu or GST-pi, with age and menopausal status of the patients, tumour size, the number of positive lymph nodes, and with cytosolic ER, PgR and cathespin D, are shown in Table II. Although no statistically significant associations (Kruskal-Wallis test) of any of the three subclasses of GST with clinical parameters were observed, GST-alpha was more often not detectable in small tumours, i.e. $T_1$-tumours were positive for GST-alpha in only four out of 46 cases (9%), as compared with 17 out of 55 (31%) $T_2$-tumours and with seven out of 28 (25%) $T_3/T_4$-tumours. Regarding an association between GST and other cytosolic factors, the highest GST-pi levels were found more frequently in ER-negative ($P = 0.05$) and PgR-negative tumours ($P < 0.03$), and in tumours with high cathepsin D concentrations, although in the latter case this association was not statistically significant. Of the ER-negative or PgR-negative tumours, 47% contained high GST-pi concentrations as compared with 26% of ER-positive or PgR-positive tumours (Table II). The associations between GST-pi and ER, PgR and cathepsin D, are visualised by scatterplots in Figure 4. A very weak negative relationship between GST-pi and ER (Spearman correlation coefficient: Rs = −0.17, $2P < 0.05$) and PgR (Rs = −0.13, N.S.) levels, and in addition a very weak positive relationship between GST-pi and cathepsin D (Rs = +0.17, $2P < 0.05$) levels were observed. GST-mu and GST-alpha concentrations were not significantly related with those of ER, PgR or cathepsin D.

### *Glutathione S-transferases and (disease-free) survival*

The levels of none of the three classes of glutathione S-transferase studied appeared to be associated with the length of disease-free survival after the administration of adjuvant chemotherapy, as is shown by Kaplan-Meier curves in Figure 5. Univariate *P*-values in Cox univariate regression analyses were 0.27, 0.24, and 0.72, respectively, for GST-alpha, -mu and -pi. Also in Cox regression analyses for overall survival, none of the GST's studied was associated with the rate of death (*P*-values ranging from 0.42 to 0.63).

**Figure 3** Scatterplot of individual values of glutathione S-transferase mu *vs.* glutathione S-transferase pi in breast tumour cytosols. Rs: Spearman rank correlation.

**Table II** Associations of GST levels with patient and tumour characteristics

| | *GST-alpha*[a] | | *GST-mu*[a] | | | *GST-pi*[a] | | |
|---|---|---|---|---|---|---|---|---|
| *Characteristic* | *Neg* | *Pos* | *Neg* | *Low* | *High* | *Low* | *Medium* | *High* |
| Number of patients[b] | 104 | 28 | 55 | 42 | 41 | 51 | 44 | 44 |
| Age | | | | | | | | |
| $\leqslant$43 yr | 40 | 10 | 22 | 14 | 15 | 15 | 18 | 18 |
| 44–50 yr | 38 | 14 | 20 | 18 | 16 | 20 | 17 | 17 |
| $\geqslant$51 yr | 26 | 4 | 13 | 10 | 10 | 16 | 9 | 9 |
| Postmenopausal | | | | | | | | |
| Yes | 15 | 3 | 7 | 8 | 5 | 11 | 5 | 5 |
| No | 89 | 25 | 48 | 34 | 36 | 40 | 39 | 39 |
| Tumour size | | | | | | | | |
| $T_1$ ($\leqslant$2 cm) | 42 | 4 | 18 | 15 | 14 | 15 | 19 | 13 |
| $T_2$ (2–5 cm) | 38 | 17 | 21 | 16 | 21 | 20 | 15 | 24 |
| $T_3$ (>5 cm) | 14 | 4 | 10 | 7 | 2 | 8 | 7 | 4 |
| $T_4$ | 7 | 3 | 6 | 3 | 3 | 7 | 2 | 2 |
| Lymph nodes | | | | | | | | |
| 1–3 | 65 | 15 | 34 | 23 | 26 | 30 | 28 | 26 |
| $\geqslant$3 | 38 | 13 | 19 | 19 | 15 | 20 | 15 | 18 |
| ER[c] | | | | | | | | |
| Neg | 27 | 6 | 15 | 14 | 6 | 8 | 11 | 17 |
| Pos | 77 | 22 | 40 | 28 | 35 | 43 | 33 | 27 |
| PgR[c] | | | | | | | | |
| Neg | 23 | 12 | 12 | 17 | 6 | 9 | 10 | 17 |
| Pos | 77 | 16 | 39 | 25 | 34 | 41 | 32 | 25 |
| Cath-D[d] | | | | | | | | |
| Low | 50 | 17 | 27 | 21 | 21 | 29 | 21 | 18 |
| High | 54 | 11 | 28 | 21 | 20 | 20 | 22 | 26 |

[a]Definition of neg, pos, low and high, are as defined in the legend to Table I. [b]Number of patients per group (due to missing values they do not all add up to 139). [c]Neg, <10; pos, $\geqslant$10 fmol mg$^{-1}$ protein. [d]Low, <45; high, $\geqslant$45 pmol mg$^{-1}$ protein.

### Discussion

Adjuvant chemotherapy is a widely used systemic treatment for breast cancer patients after surgical removal of the tumour. However, in the vast majority of the cases resistance to this therapy develops in the treatment of metastatic disease. Understanding of the mechanism underlying this resistance should lead to future improvements of therapeutic results. A wide variety of factors may be involved in resistance to chemotherapeutics of breast tumours, and recent evidence suggests that glutathione S-transferase pi may be of relevance (Cowan *et al.*, 1986; Moscow *et al.*, 1988). Having studied specimens from 21 breast cancer patients, Moscow *et al.* reported an inverse relationship between glutathione S-transferase pi levels and oestrogen receptor content, a finding which was confirmed by Howie *et al.* (1989) in 58 patients, but not by Shea *et al.* (1990), who investigated 45 tumour samples. In this study involving 139 different tumour samples, also a weak but statistically significant inverse correlation between GST-pi and ER was found. In addition, we observed a weak inverse correlation between GST-pi and PgR, and a weak positive correlation between GST-pi and cathepsin D.

Class mu glutathione S-transferase deficiency, occurring in

**Figure 4** Scatterplots of associations of glutathione S-transferase pi with ER **a**, PgR **b** and cathepsin D **c**. Rs: Spearman rank correlation.

**Figure 5** Probability disease-free survival curves stratified by glutathione S-transferase class alpha **a**, mu **b** and pi status **c**. **a**, —— negative (53/104), --- positive (11/28); **b**: —— negative 24/55), --- low (22/42), — high (20/41); **c**: —— low (24/51), --- medium (22/44), — high (19/44). Numbers between parentheses represent failures (occurring in the first 60 months)/total number of patients in each group. Negative, low, medium and high for glutathione S-transferase mu or pi are as defined in the legend to Table I.

approximately 40% of a normal Caucasian population has been implicated with an increased risk for developing lung carcinomas in smokers (Seidegard *et al.*, 1990). In addition, increased cytogenetic damage was observed in *in vitro* studies with glutathione S-transferase mu deficient human blood cells (Wiencke *et al.*, 1990; van Poppel *et al.*, 1992). Our results demonstrate that 40% of the 138 breast cancer samples investigated are negative for the mu class enzymes. So in accordance with an earlier study involving a different group of 52 breast cancer patients (Peters *et al.*, 1990a) this more extensively study leads to the same conclusion that glutathione S-transferase mu deficiency does not occur more often in breast cancer patients and therefore class mu deficiency seems not to be involved in the aetiology of breast cancer.

The disease-free survival curves for our patient group treated with adjuvant chemotherapy show an equal relapse pattern for tumours with a high or low content of glutathione S-transferase alpha, mu or pi. Therefore these glutathione S-transferases are not useful as predictive markers for patients to be treated with adjuvant chemotherapy. Adjuvant chemotherapy with CMF is especially effective in premenopausal patients but also significantly in the age group of 50–60 years-old (Early Breast Cancer Trialists' Collaborative Group, 1992), to which category a small minority of our patients belong. The higher efficacy in younger patients compared to patients over 60 years of age is possibly related to an endocrine mechanism of action, namely chemical castration. Whether the glutathione S-transferases are relevant in patients receiving hormonal therapy or no treatment needs to be investigated. In a very preliminary study on 34 patients with ER-positive locally advanced breast cancer, Dorian-Bonnet *et al.* (1992) found that 12 patients with an objective response to tamoxifen showed significant lower GST-pi levels in their tumour than the other non-responding patients. Our results are in good agreement with a recent study in 68 patients with advanced breast cancer receiving mitozantrone therapy. In this study of Wright *et al.* (1992), using an immunohistochemical method to assess GST status, no correlation between glutathione S-transferase alpha, mu or pi content of the primary tumour and the response rate or duration of response could be detected. Also in an *in vitro* chemosensitivity study on primary breast cancer tissue from untreated patients, no correlation between drug (doxorubicin) sensitivity and glutathione S-transferase (pi) was observed (Keith *et al.*, 1990). For patients with ovarian cancer, large changes in glutathione S-transferase enzyme activity or isoenzyme (alpha, mu and pi) expression were not likely to be a major determinant of resistance to chemotherapy (Murphy *et al.*, 1992). In addition, a lack of a role of glutathione S-transferase pi in drug resistance of malignant ovarian tumours to platinum/cyclophosphamide chemotherapy has recently been reported (van der Zee *et al.*, 1992).

In contrast to many other tumour types such as tumours from the lung (Howie *et al.*, 1990), stomach (Peters *et al.*, 1990*b*), colon (Peters *et al.*, 1992), or ovary (Murphy *et al.*, 1992), breast tumours do not express higher levels of glutathione S-transferases as compared to their corresponding normal tissues (Howie *et al.*, 1990: Tsuchida & Sato, 1992). Thus the transformed breast cell in this respect is similar to the normal breast cell which also argues against a role for glutathione S-transferase with respect to malignant transformation and drug resistance of the breast (tumour) cells.

In conclusion, the lack of an association of glutathione S-transferases with the length of disease-free survival following adjuvant CMF chemotherapy of patients with primary breast cancer, suggests that these detoxifying enzymes are not useful in selecting patients who may benefit from adjuvant CMF chemotherapy. In view of the fact that no control group was included in this study, a possible prognostic (instead of predictive) value of glutathione S-transferases in an untreated patient population cannot totally be excluded.

We are indebted to Drs Y.W.C.M. de Koning, J. Alexieva-Figusch and M. Bontenbal, for carefully collecting the follow-up data of the patients, and to Mr H. Portengen, H.A. Peters and P. van Assendelft for expert technical assistance. This study was supported by the Dutch Cancer Society (Grant DDHK 92-04).

### References

BOLTON, M.G., COLVIN, O.M. & HILTON, J. (1991). Specificity of isoenzymes of murine hepatic glutathione S-transferase for the conjugation of glutathione with L-phenylanaline mustard. *Cancer Res.*, **51**, 2410–2415.

CAMPBELL, J.A.H., CORRIGALL, A.V., GUY, A. & KIRCH, R.E. (1991). Immunohistologic localization of alpha, mu, and pi class glutathione S-transferases in human tissues. *Cancer*, **67**, 1608–1613.

CAZENAVE, L.A., MOSCOW, J.A., MYERS, C.E. & COWAN, K.H. (1989). Glutathione S-transferase and drug resistance. In *Drug Resistance in Cancer Therapy*. Ozols, R.F. (ed.), pp. 171–187, Kluwer Academic Publishers, Boston, M.A., USA.

CIACCIO, P.J., TEW, K.D., & LACRETA, F.P. (1991). Enzymatic conjugation of chlorambucil with glutathione by human glutathione S-transferases and inhibition by ethacyrnic acid. *Biochem. Pharmacol.*, **42**, 1504–1507.

COWAN, K.H., BATIST, G., TULPULE, A., SINHA, B.K. & MYERS, C.E. (1986). Similar biochemical changes associated with multidrug resistance in human breast cancer cells and carcinogen-induced resistance to xenobiotics in rats. *Proc. Natl Acad. Sci. USA*, **83**, 9328–9332.

DORIAN-BONNET, F., QUENEL, N., COINDRE, J.M., MAURIAC, L., BONICHON, F., DURAND, M., MOSCOW, J.A., COWAN, K.H. & GUALDE, N. (1992). Expression of the GST-pi gene and response to tamoxifen therapy in locally advanced breast carcinomas. Pisa Symposia in Oncology. *Breast Cancer: from Biology to Therapy*, Pisa, October 19–21, 1992, p54, abstract 17.

DULIK, D., FENSELAU, C. & HILTON, J. (1986). Characterization of melphalanglutathione adducts whose formation is catalyzed by glutathione S-transferases. *Biochem. Pharmacol.*, **35**, 3405–3409.

EARLY BREAST CANCER TRIALISTS' COLLABORATIVE GROUP (1992). Systemic treatment of early breast cancer by hormonal, cytotoxic, or immune therapy. *Lancet*, **339**, 1–15, 71–85.

FORTC BREAST CANCER COOPERATIVE GROUP (1980). Revision of the standards for the assessment of hormone receptors in human breast cancer. *Eur. J. Cancer*, **16**, 1513–1515.

FOEKENS, J.A., PORTENGEN, H., VAN PUTTEN, W.L.J., TRAPMAN, A.M.A.C., REUBI, J.-C., ALEXIEVA-FIGUSCH, J. & KLIJN, J.G.M. (1989*b*). Prognostic value of receptors for insulin-like growth factor 1, somatostatin, and epidermal growth factor in human breast cancer. *Cancer Res*, **49**, 7002–7009.

FOEKENS, J.A., PORTENGEN, H., VAN PUTTEN, W.L.J., PETERS, H.A., KRIJNEN, H.L.J.M., ALEXIEVA-FIGUSCH, J. & KLIJN, J.G.M. (1989*b*). Prognostic value of estrogen and progesterone receptors measured by enzyme immunoassays in human breast tumor cytosols. *Cancer Res.*, **49**, 5823–5828.

FORRESTER, L.M., HAYES, J.D., MILLIS, R., BARNES, D., HARRIS, A.L., SCHLAGER, J.J., POWIS, G. & WOLF, C.R. (1990). Expression of glutathione S-transferases and cytochrome P-450 in normal and tumor breast tissue. *Carcinogenesis*, **11**, 2163–2170.

HARRIS, A.L. (1990). Mechanisms of anticancer drug resistance. In *Glutathione S-transferases and Drug Resistance*. Hayes, J.D., Pickett, C.B. & Mantle, T.J. (eds) pp. 283–293, Taylor and Francis, London.

HOWIE, A.F., MOLLER, W.R., HAWKINS, R.A., HUTCHINSON, A.R. & BECKETT, G.J. (1989). Expression of glutathione S-transferase B1, B2, mu and pi in breast cancers and their relationship to oestrogen receptor status. *Br. J. Cancer*, **60**, 834–837.

HOWIE, A.F., FORRESTER, L.M., GLANCEY, M.J., SCHLAGER, J.J., POWIS, G., BECKETT, G.J., HAYES, J.D. & WOLF, C.R. (1990). Glutathione S-transferase and glutathione peroxidase expression in normal and tumour human tissues. *Carcinogenesis*, **11**, 451–458.

KANTOR, R.R.S., GIARDINA, S.L., BARTOLAZZI, A., TOWNSEND, A.J., MYERS, C.E., COWAN, K.H., LONGO, D.L. & NATALI, P.G. (1991). Monoclonal antibodies to glutathione S-transferase pi. Immunohistochemical analysis of human tissues and cancers. *Int. J. Cancer*, **47**, 193–201.

KEITH, W.N., STALLARD, S. & BROWN, R. (1990). Expression of MDR1 and GST-pi in human breast tumours: comparison to *in vitro* chemosensitivity. *Br. J. Cancer*, **61**, 712–716.

MANNERVIK, B. & DANIELSON, U.H. (1988). Glutathione S-transferases, structure and catalytic activity. *CRC Crit. Rev. Biochem.*, **23**, 283–337.

MOSCOW, J.A., TOWNSEND, A.J., GOLDSMITH, M.E., WHANG-PENG, J., VICKERS, P.J., POISSON, R., LEGAULT-POISSON, S., MYERS, C.E. & COWAN, K.H. (1988). Isolation of the human anionic glutathione S-transferase cDNA and the relation of its gene expression to estrogen-receptor content in primary breast cancer. *Proc. Natl Acad. Sci. USA*, **85**, 6518–6522.

MURPHY, D., MCGOWN, A.T., HALL, A., CATTAN, A., CROWTHER, D. & FOX, B.W. (1992). Glutathione S-transferase activity and isoenzyme distribution in ovarian tumour biopsies taken before or after cytotoxic chemotherapy. *Br. J. Cancer*, **66**, 937–942.

NAMER, M., RAMAIOLI, A., FONTANA, X., ETIENE, M.-C., 'HÉRY, M., JOURLAIT, A., MILANO, G., FRENAY, M., FRANCOIS, E. & LAPALUS, F. (1991). Prognostic value of total cathepsin D in breast tumors. *Breast Cancer Res. Treatm.*, **19**, 85–93.

PETERS, W.H.M. & JANSEN, P.L.M. (1988). Immunocharacterization of UDP-glucuronyltransferase isoenzymes in human liver, intestine and kidney. *Biochem. Pharmacol.*, **37**, 564–567.

PETERS, W.H.M., NAGENGAST, F.M. & WOBBES, TH. (1989). Glutathione S-transferases in normal and cancerous human colon tissue. *Carcinogenesis*, **10**, 2371–2374.

PETERS, W.H.M., KOCK, L., NAGENGAST, F.M. & ROELOFS, H.M.J. (1990*a*). Immunodetection with a monoclonal antibody of glutathione S-transferase mu in patients with and without carcinomas. *Biochem. Pharmacol.*, **39**, 591–597.

PETERS, W.H.M., WORMSKAMP, N.G.M. & THIES, E. (1990*b*). Expression of glutathione S-transferases in normal gastric mucosa and in gastric tumors. *Carcinogenesis*, **11**, 1593–1596.

PETERS, W.H.M., BOON, C.E.W., ROELOFS, H.M.J., WOBBES, T.H., NAGENGAST, F.M. & KREMERS, P.G. (1992). Expression of drug-metabolizing enzymes and P-170 glycoprotein in colorectal carcinoma and normal mucosa. *Gastroenterol.*, **103**, 448–455.

ROCHEFORT, H. (1992). Cathepsin D in breast cancer: a tissue marker associated with metastasis. *Eur. J. Cancer*, **28A**, 1780–1783.

SEIDEGARD, J., PERO, R.W., MARKOWIK, M.M., ROUSH, G., MILLER, D.G. & BEATTIE, E.J. (1990). Isoenzymes of glutathione S-transferase (class mu) as a marker for susceptibility to lung cancer: a follow up study. *Carcinogenesis*, **11**, 33-36.

SHEA, T.C., CLAFLIN, G., COMSTOCK, K.E., SANDERSON, B.J.S., BURSTEIN, N.A., KEENAN, E.J., MANNERVIK, B. & HENNER, W.D. (1990). Glutathione transferase activity and isoenzyme composition in primary human breast cancers. *Cancer Res.*, **50**, 6848-6853.

TERRIER, P., TOWNSEND, A.J., COINDRE, J.M., TRICHE, T.J. & COWAN, K.H. (1990). An immunohistochemical study of pi class glutathione S-transferase expression in normal human tissue. *Am. J. Pathol.*, **137**, 845-853.

TSUCHIDA, S. & SATO, K. (1992). Glutathione transferases and cancer. *CRC Crit. Rev. Biochem. Mol. Biol.*, **27**, 337-384.

VAN DER ZEE, A.G.J., VAN OMMEN, B., MEIJER, C., HOLLEMA, H., VAN BLADEREN, P.J. & DE VRIES, E.G.E. (1992). Glutathione S-transferase activity and isoenzyme composition in benign ovarian tumours, untreated malignant ovarian tumours, and malignant ovarian tumours after platinum/cyclophosphamide chemotherapy. *Br. J. Cancer*, **66**, 930-936.

VAN POPPEL, G., DE VOGEL, N., VAN BLADEREN, P.J. & DE KOK, F.J. (1992). Increased cytogenetic damage in smokers deficient in glutathione S-transferase isoenzyme mu. *Carcinogenesis*, **13**, 303-305.

VOS, R.M.E. & VAN BLADEREN, P.J. (1990). Glutathione S-transferases in relation to their role in the biotransformation of xenobiotics. *Chem. Biol. Interact.*, **75**, 241-265.

WAXMAN, D.J. (1990). Glutathione S-transferase: role in alkylating agent resistance and possible target for modulation chemotherapy - a review. *Cancer Res.*, **50**, 6449-6454.

WIENCKE, J.K., KELSEY, K.T., LAMELA, R.A. & TOSCANO, W.A. (1990). Human glutathione S-transferase deficiency as a marker of susceptibility to epoxide induced cytogenetic damage. *Cancer Res.*, **50**, 1585-1590.

WOLF, C.R., MCPHERSON, J.S. & SMYTH, J.F. (1986). Evidence for the metabolism of mitozantrone by microsomal glutathione transferases and 3-methylcholanthrene-inducible glucuronosyltransferases. *Biochem. Pharmacol.*, **35**, 1577-1581.

WRIGHT, C., CAIRNS, J., CANTWELL, B.J., CATTAN, A.R., HALL, A.G., HARRIS, A.L. & HORNE, C.H.W. (1992). Response to mitozantrone in advanced breast cancer: correlation with expression of c-erbβ-2 protein and glutathione S-transferases. *Br. J. Cancer*, **65**, 271-274.

YUAN, Z., SMITH, P.B., BRUNDRETT, R.B., COLVIN, M. & FENSELAU, C. (1991). Glutathione conjugation with phosphoramide mustard and cyclophosphamide. *Drug Metab. Dispos.*, **19**, 625-629.